# Schizophrenia And Polycystic Ovary Syndrome: A Case Report

**DOI:** 10.1192/j.eurpsy.2022.2028

**Published:** 2022-09-01

**Authors:** M. Turki, O. Abidi, S. Ellouze, F. Ben Abdallah, F. Youzbechi, N. Halouani, J. Aloulou

**Affiliations:** Hedi Chaker University Hospital, Psychiatry “b” Department, Sfax, Tunisia

**Keywords:** comorbidity, schizophrénia, polycistic ovary syndrome

## Abstract

**Introduction:**

Patients with Polycystic ovary syndrome (PCOS) have increased vulnerability to psychiatric disorders, particularly a tendency to depression and anxiety, as well as schizophrenia. The association between PCOS and psychiatric disorders is a topic of research given the possibility of common potential mechanisms as well as the clinical similarity between the adverse effects of atypical antipsychotics and the symptoms of PCOS.

**Objectives:**

We proposed to investigate the etiopathogenic relationship between schizophrenia and PCOS as well as the therapeutic particularities.

**Methods:**

We report a case of schizophrenia occurring in a patient with PCOS. Then, we conducted a literature review using “PubMed” database and keywords “psychosis”, “schizophrenia”, “Polycystic ovary syndrome” and “antipsychotic drugs”.

**Results:**

She was an 18-year-old patient, diagnosed with PCOS since 2018. She has been followed in the psychiatry outpatient department since 8 months for psychotic symptoms (hallucinatory syndrome with thoughts of self-aggressiveness, delusional syndrome with mental automatism…). She was prescribed olanzapine (5 then 10 mg/day). However, after a weight gain (4 kg per month), this drug was switched by Risperidone (2 then 4 mg/day). The evolution was marked by the appearance of galactorrhea. Thus, the Risperidone was switched to Aripiprazole. Then, we noted a significant improvement on the psychiatric features and a better clinical tolerance.

**Conclusions:**

For women with PCOS and psychosis, treatment with antipsychotic drugs can worsen PCOS symptomatology and lead to negative consequences for a woman’s reproductive potential and her quality of life. Therefore, the psychosis management must take these particularities into account, in order to improve the prognosis of both diseases.

**Disclosure:**

No significant relationships.

